# Wastewater-Based Surveillance of Respiratory Viruses in the SARS-CoV-2 Post-Pandemic Period in Mexico

**DOI:** 10.3390/v18020254

**Published:** 2026-02-17

**Authors:** Oscar Uriel Ulloa-Medina, Pedro Gerardo Hernández-Sánchez, Gabriel Mata-Moreno, Luis Rubén Jaime-Rocha, Sara del Carmen Alatorre-Camacho, Ignacio Lara-Hernández, Mauricio Comas-García, Juan Carlos Muñoz-Escalante, Ana María González-Ortiz, Pedro Torres-González, Daniel E. Noyola

**Affiliations:** 1 Centro de Investigación en Ciencias de la Salud y Biomedicina, Universidad Autónoma de San Luis Potosí, Av. Sierra Leona 550, Lomas de San Luis, San Luis Potosí 78210, SLP, Mexico; odeoscar9@gmail.com (O.U.U.-M.); pedro.hernandez@uaslp.mx (P.G.H.-S.); gabriel.mata@uaslp.mx (G.M.-M.); carlos.escalante@uaslp.mx (J.C.M.-E.); pedrotorresgonzalez@gmail.com (P.T.-G.); 2 Facultad de Medicina, Universidad Autónoma de San Luis Potosí, Av. Venustiano Carranza 2405, Lomas los Filtros, San Luis Potosí 78210, SLP, Mexico; 3 Facultad de Ciencias, Universidad Autónoma de San Luis Potosí, Av. Parque Chapultepec 1570, Privadas del Pedregal, San Luis Potosí 78295, SLP, Mexico; 4 Hospital de la Niñez y la Mujer “Dr. Alberto López Hermosa”, Blvd. Antonio Rocha Cordero 2510, San Juan de Guadalupe, San Luis Potosí 78364, SLP, Mexico; anagon71@yahoo.com.mx

**Keywords:** wastewater, wastewater-based epidemiology, SARS-CoV-2, influenza, respiratory syncytial virus, human metapneumovirus, COVID-19, infectious diseases, surveillance, environmental surveillance

## Abstract

In recent years, wastewater (WW)-based epidemiology has been increasingly used for surveillance of SARS-CoV-2 and has emerged as a potential tool for monitoring other respiratory viruses. Most evidence on the use of WW for detecting multiple respiratory viruses comes from developed countries. In this study, we assessed the feasibility of multi-respiratory virus sewage surveillance in a middle-income country and explored signals that may be potentially used as early warning signs for Public Health authorities. We examined the presence of SARS-CoV-2, influenza virus, respiratory syncytial virus (RSV), and human metapneumovirus (hMPV) in 238 WW samples collected from three treatment plants in San Luis Potosí, Mexico, over one year. The weekly detection of each virus was compared with the weekly number of hospital admissions for respiratory infections caused by that virus in pediatric patients. SARS-CoV-2, influenza, hMPV, and RSV were detected in 152 (63.9%), 108 (45.4%), 95 (39.9%), and 24 (10.1%) samples, respectively. There was no significant correlation between viral detection in WW and the number of hospitalizations during that week. However, analyses of WW viral detection with hospitalizations in subsequent weeks showed an increasing correlation reaching a maximum correlation for a lag of 12 weeks for SARS-CoV-2 (r_s_ = 0.63, *p* = 0.001), 9 weeks for influenza (r_s_ 0.62, *p* = 0.0001), 2 weeks for RSV (r_s_ = 0.30, *p* = 0.05), and 3 weeks for hMPV (r_s_ = 0.39, *p* = 0.009). In addition, we identified time-periods of SARS-CoV-2, influenza, and RSV widespread circulation (several consecutive weeks in which viruses were detected in the three treatment plants); most hospitalizations caused by these viruses occurred after widespread circulation was detected in WW, suggesting this may be used as an early alert for public health systems. Overall, our results show that WW-based surveillance of multiple respiratory viruses is feasible and has potential applications as an early warning system in middle-income countries.

## 1. Introduction

Respiratory viruses are a leading cause of respiratory infections worldwide. Most countries experience seasonal patterns of acute respiratory infections that lead to hospitalization, with cases peaking in the fall and winter [1]. This increase is primarily due to the epidemic spread of certain viruses during winter, typically influenza and respiratory syncytial virus (RSV). However, viruses such as human metapneumovirus (hMPV) and, more recently, SARS-CoV-2 are now contributing to the seasonal burden of hospitalizations for acute respiratory infections.

Although this seasonal trend is well recognized, the timing of epidemics and the predominant respiratory virus can vary from year to year, and disruptions to expected seasonality may occur due to the emergence of new pathogens, as observed during the SARS-CoV-2 pandemic [2].

Virological surveillance is an important tool for the timely identification of acute respiratory infection epidemics and for monitoring the emergence of potential public health threats [3]. However, most epidemiological surveillance systems rely on identifying patients who seek medical care and meet specific case definitions, as well as performing viral diagnostic testing on samples obtained from these patients. These systems require healthcare workers to actively identify which patients should be tested, and samples from all patients (or those identified at specific sites in sentinel surveillance systems) must be submitted to the laboratory for viral testing [4]. Therefore, active involvement of healthcare workers across medical units is required, and public health laboratories may be overwhelmed and face many challenges in conducting testing during major epidemics, as observed during the COVID-19 pandemic [5,6]. During recent years, wastewater (WW)-based epidemiological surveillance has gained attention as a complementary tool to established surveillance systems [7]. As a result, SARS-CoV-2 surveillance using WW samples is routinely conducted in many countries [8]. However, comparing results across systems in different countries or within the same country across cities is not always feasible because many factors can influence outcomes. These factors include the equipment used for sample processing, virus detection protocols, sewage network organization, population density, and the presence of industrial waste [9,10]. In addition, information regarding WW surveillance for respiratory viruses other than SARS-CoV-2 is limited and has mostly been obtained in developed countries. Moreover, although for some viruses, such as SARS-CoV-2, the correlation of the detection of the virus in WW and their circulation in the community is well established (and may even predict the rise in cases in the population), whereas for other respiratory viruses (hMPV, RSV), it is not [11,12,13,14]. Based on this, we conducted a study to assess the feasibility of using WW samples for epidemiological surveillance of respiratory viruses, including SARS-CoV-2, influenza, RSV, and hMPV, in San Luis Potosí, Mexico. Here, we report results from samples collected at three WW treatment plants (WWTPs) over a one-year period. The objectives of our study were to (a) verify the feasibility of multi-respiratory virus sewage surveillance in a middle-income country, and (b) explore signals that may be potentially used as early warning signs for Public Health authorities (such as extent of viral circulation, and lagged correlation analyses between WW viral detection and pediatric hospitalizations).

## 2. Materials and Methods

### 2.1. Location and Samples

This study was conducted in San Luis Potosí, Mexico, located in central-northern Mexico, with a population of approximately 1.24 million. Samples were collected from three WWTPs serving the metropolitan area of San Luis Potosí, including the municipalities of San Luis Potosí and Soledad de Graciano Sánchez. Composite 24-h inflow samples were taken twice per week (Tuesdays and Thursdays) from each WWTP, named Tangamanga 1, Tangamanga 2, and Norte; hereafter referred to as P1, P2, and PN. These WWTP collectively process about 75% of the city’s population. The samples were transported under refrigerated conditions to our laboratories at the Centro de Investigación en Ciencias de la Salud y Biomedicina (Universidad Autónoma de San Luis Potosí) for analysis. Samples collected over a one-year period, from epidemiological week 24 of 2024 to week 24 of 2025, were analyzed.

### 2.2. Sample Processing

Five hundred mL of WW samples were collected using polypropylene bottles. Due to administrative adjustments, no samples were collected from epidemiological week 51 of 2024 through week 4 of 2025. Following transportation, samples were thermally inactivated in a water bath at 65 °C for 30 min to ensure safe handling. After incubation, samples were stored at −20 °C until further processing [15].

Viral genomic material was concentrated by ultracentrifuging 100 mL of inactivated WW samples at 141,000× *g* for 4 h using an Optima XE-100 ultracentrifuge with an SW28 rotor (Beckman Coulter, CA, USA) [16]. The resulting pellet was resuspended in PBS 1× a final volume of 200 µL. Concentrated samples were then stored in 2.5 mL tubes at 4 °C until use.

### 2.3. RNA Extraction, cDNA Synthesis, and Viral Detection

RNA was extracted from the concentrated WW obtained after ultracentrifugation using the RNeasy PowerWater Kit (QIAGEN, Hilden, Germany) according to the manufacturer’s instructions, yielding a final volume of 100 µL of RNA. Extracted RNA samples were stored at −80 °C until use. Complementary DNA (cDNA) was synthesized from the RNA using the RevertAid H Minus First Strand cDNA Synthesis Kit (ThermoFisher Scientific, Waltham, MA, USA) with random primers, according to the manufacturer’s instructions.

Detection of SARS-CoV-2, influenza A, influenza B, RSV, hMPV, and crAssphage (as human fecal marker and amplification control) was performed using virus-specific quantitative PCR (qPCR) assays with Maxima SYBR Green/Rox qPCR Master Mix (ThermoFisher Scientific, Waltham, MA, USA). Previously described primers were used for detection [17,18,19,20,21,22]. To assess the potential contribution of avian influenza viruses, a subset of influenza A-positive WW samples was further tested for influenza A H5 and H7 [23]. Primer sequences and amplification conditions are detailed in Tables S1–S3. Absolute quantification of viral copy numbers was performed using standard curves generated from synthetic plasmids containing the corresponding qPCR target sequences. Standard curves were established from serial dilutions of plasmids with known concentrations, and amplification efficiency and linearity were verified for each assay. Viral genomes copy numbers in WW samples were calculated from the standard curve regression equation, using the cycle threshold values obtained for each sample, and expressed as viral copies per liter of WW. Influenza virus circulation was analyzed based on the combined results of influenza A and B detections.

To assess recovery and process performance, preliminary spiking experiments were conducted using WW samples previously tested negative for RSV. Known concentrations of quantified RSV-positive material or synthetic DNA controls targeting the RSV qPCR assay were added prior to sample processing (before heat inactivation). Detected concentrations after the complete workflow (heat inactivation, ultracentrifugation, RNA extraction, cDNA synthesis, and qPCR) were consistent with the expected values based on dilution factors. Only the samples from which crAssphage was amplified were considered for the analysis of the rest of the viruses.

Concentrated samples were aliquoted to allow up to three independent RNA extractions; a single extraction was performed per sample. All qPCR detections and absolute quantifications were performed in technical duplicates. Standard curve points were also run in duplicate, and only assays meeting predefined quality criteria (R2 > 0.98) were considered valid.

### 2.4. Epidemiological Surveillance

In order to assess the results from viral testing in WW samples and their relationship with the disease burden in the population, we used data from a surveillance system, which we have previously established at a public hospital in San Luis Potosí (Hospital de la Niñez y la Mujer “Dr. Alberto López Hermosa”) [24]. Children admitted to the hospital with lower respiratory tract infections were enrolled in the surveillance study and respiratory samples were tested to detect viruses, including SARS-CoV-2, influenza, RSV, and hMPV. Sample collection and viral testing are part of a research project approved by the Research and Ethics Committee of the Ministry of Health of the Government of the State of San Luis Potosí (approval number SLP/04-2023), and informed consent from parents was obtained prior to sample collection. The weekly counts of admissions and detections for each virus were used as a proxy for viral circulation in the population. Of note, the weekly number of admissions correlated with the number of pneumonia cases for all ages reported by the Health Ministry for San Luis Potosí [25] (Figure S1).

### 2.5. Statistical Analysis

Before data analysis, the viral concentration (copies/L) obtained from the standard curve of each virus was normalized for the fecal concentration (e.g., RSV copies/L divided by the crAssphage copies/L), and for the average week reading of each virus the samples from the same epidemiological week were weighted by the WWTP inflow (P1: 110 L per second; P2: 40 L per second; PN: 400 L per second), afterwards averaged, and finally transformed to log10 and multiplied by 10,000 to facilitate data management. Descriptive analysis includes frequencies (numbers and proportions) for qualitative data and means (and standard deviations) for quantitative data (log-transformed virus concentrations). Comparisons were carried out using chi-squared or Fisher’s exact test for qualitative data and Mann–Whitney U or Student’s *t* test for quantitative data. Correlations were assessed using the Spearman correlation test. Correlations were carried out comparing WW viral concentrations and number of hospitalizations during the same week; in addition, comparisons between WW viral concentrations with admissions during subsequent weeks with lag 1 to 14 weeks were also performed. A *p*-value < 0.05 was considered statistically significant. Analysis was performed using OpenEpi (OpenEpi: Open Source Epidemiologic Statistics for Public Health, Version. www.OpenEpi.com, accessed on 5 January 2026) and Stata 18 (StataCorp LLC, College Station, TX, USA).

## 3. Results

During the one-year period, we collected 238 WW samples. The six samples scheduled for each week (two per treatment plant) were obtained in 29 weeks, while only five samples were obtained in five weeks, three samples in 13 weeks, and there were six weeks in which no samples were available due to administrative adjustments at the WWTP (week 51 2024, through week 4 2025).

**Figure 1:**
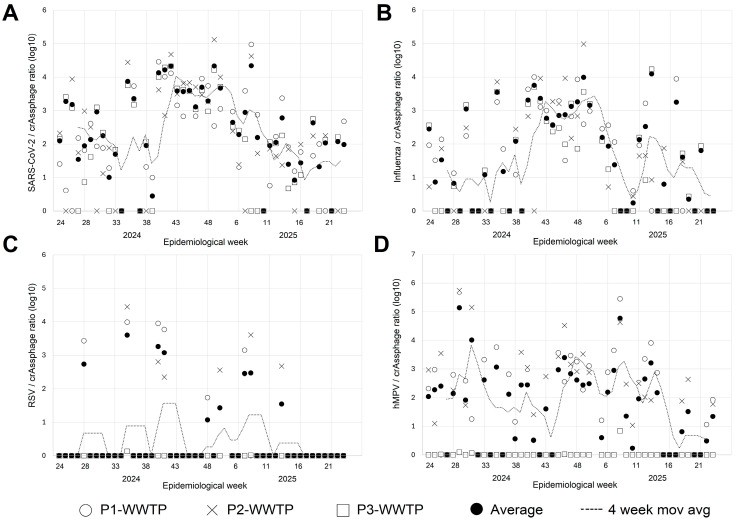
Weekly mean copy number of SARS-CoV-2 (**A**), influenza (**B**), RSV (**C**), and hMPV (**D**) in wastewater in San Luis Potosí, Mexico, from epidemiological week 24, 2024, to epidemiological week 24, 2025. The average concentration in each of the WWTP (Tangamanga 1 (P1), Tangamanga 2 (P2), and Norte (PN)) as well as the average is shown for each week. Dashed lines show the 4-week moving average of the average viral concentration in WW.

SARS-CoV-2 was detected in 152 samples, influenza virus (A or B) in 108, RSV in 24, and hMPV in 95. To assess the potential contribution of avian influenza viruses, a subset of influenza A-positive WW samples was further tested for influenza A H5 and H7; all tested samples were negative. In addition, no avian influenza cases were reported in the study area during the study period. Analysis of the weekly results at each WWTP showed that SARS-CoV-2, influenza, and hMPV were detected in most weeks across all three WWTP, while RSV was detected less frequently (Table 1). The weekly average adjusted copy number of each virus at each WWTP and weighted combined average of all WWTPs is shown in Figure 1.

**Figure 2:**
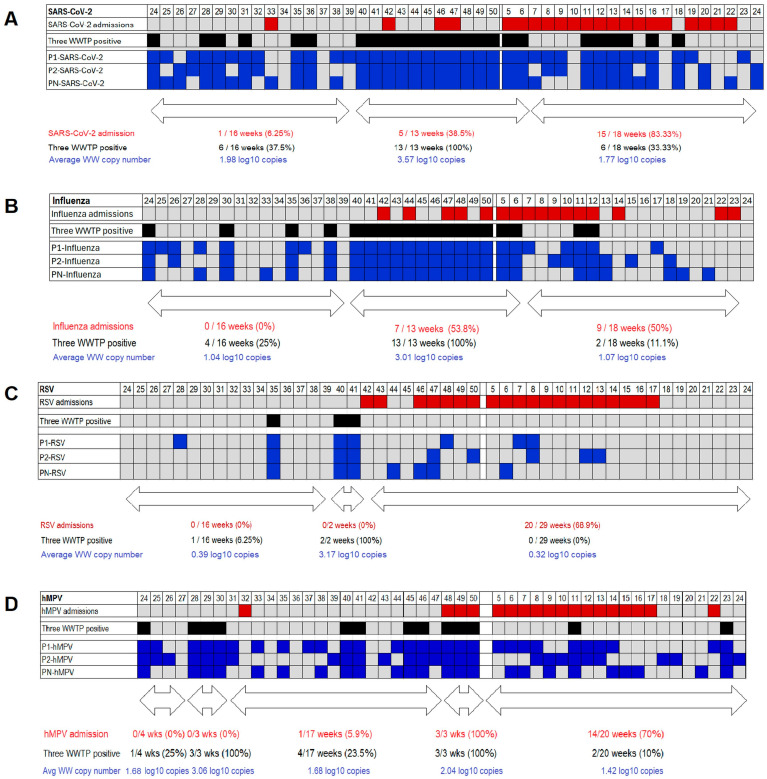
SARS-CoV-2 (**A**), influenza (**B**), RSV (**C**), and hMPV (**D**) detection in each WWTP (blue squares), detection in all three WW treatment plants (black squares), and hospital admissions (red squares) during each week. For SARS-CoV-2, influenza, and RSV, the study year is divided into three time periods as follows: (1) period before continuous widespread circulation of a virus is detected; (2) period of continuous widespread circulation of a virus; and (3) period after widespread circulation of the virus stops. For hMPV, the study year is divided into five periods as follows: (1) period before continuous widespread circulation of hMPV; (2) first period of widespread circulation of hMPV; (3) period after widespread circulation of hMPV; (4) second period of widespread circulation of hMPV; (5) period after second widespread circulation of hMPV. Arrow-headed lines indicate the duration of the above-mentioned time periods for each virus. No WW samples were available for weeks 51 and 52 of 2024 and weeks 1 to 4 of 2025.

Overall, there was a strong correlation in weekly viral detection across the treatment plants (Table 2). The Spearman correlation coefficient ranged from 0.33 for comparisons of hMPV concentrations between P1-WWTP and P2-WWTP to 0.99 for RSV concentrations between P2-WWTP and PN-WWTP. However, viruses were detected in only one or two WWTPs in some weeks. Of interest, in the case of SARS-CoV-2 and influenza most weeks in which these viruses were detected in the three WWTP were consecutive weeks between epidemiological week 40 of 2024 and epidemiological week 6 of 2025 (Figure 2). In addition, we noted that prior to the start of this continuous detection of SARS-CoV-2 and influenza in all three WWTP there were no influenza admissions and SARS-CoV-2 admissions occurred only in one week, while most hospital admissions occurred after the continuous period of viral detection in the three WWTP. Detections of RSV in WW were more sporadic, but we observed that there were two consecutive weeks in which this virus was detected in the three WWTP (epidemiological weeks 40 and 41) and all RSV admissions occurred after these weeks. Detection of hMPV in WW also occurred mostly in one or two WWTP during a given week, but a distinct period in which this virus was detected in all three WWTP was not identified, since there were two three-week periods with this characteristic.

There were 330 hospital admissions due to lower respiratory tract infections at the surveillance hospital during the study period; the most frequently detected virus was RSV (172 cases, 52%), followed by hMPV (119 cases, 36%), SARS-CoV-2 (115 cases, 35%), and influenza (68 cases, 21%). Two hundred and fifty-one of these patients were admitted during the weeks for which WW samples were available, and the number of viral detections during those weeks was 115 for RSV, 95 for hMPV, 78 for SARS-CoV-2, and 39 for influenza.

SARS-CoV-2 was detected in samples from at least one WWTP in 42 of 47 weeks (89.4%). Simultaneous detection in all three WWTP was recorded in 25 (58.1%) weeks in which the virus was detected, while detection in two and one WWTP occurred in 12 and 5 weeks, respectively (Table 2). There was no association between detection of SARS-CoV-2 in WW and hospital admissions when the virus was identified during that week (Spearman’s r = −0.22; *p* = 0.13). No association between the number of WWTPs positive for SARS-CoV-2 and the average number of admissions was observed (Table 3). Of interest, most weeks in which SARS-CoV-2 was detected in all three WWTP clustered in a continuous period between epidemiological week 40 of 2024 and week 6 of 2025 (Figure 2). Average SARS-CoV-2 copy number in WW was higher during that period than during the period before (weeks 24 to 39, 2024) and after (weeks 7 to 24, 2025) that period (*p* < 0.001 for both comparisons). In contrast, there was no difference in the WW copy number of SARS-CoV-2 between the periods before and after this continuous, widespread SARS-CoV-2 detection (*p* = 0.60). Only two SARS-CoV-2-associated admissions during a single week were recorded during the first period (weeks 25 to 39, 2024) (admissions recorded in 1 of 16 weeks, 6.25%), while SARS-CoV-2-associated admissions started to increase during the widespread circulation period (admissions recorded in 5 of 13 weeks, 38.55%). Interestingly, SARS-CoV-2-associated admissions were even more frequently detected during the third period (admissions recorded in 15 of 18 weeks, 83.33%), exhibiting an important lag between virus detection in WW and hospital admissions. To assess this, we analyzed the correlation between WW virus concentrations in a given week with the number of hospitalizations in subsequent weeks (lag ranging from 1 to 14 weeks) (Table S4). A progressive increase in correlation was observed with increasing lag between the week of WW SARS-CoV-2 concentration measurement and the week of hospital admission, with the highest correlation at a 12-week lag (Spearman’s r = 0.63; *p* = 0.001).

Influenza virus was also detected in WW in most weeks (33 of 47, 70.2%). Simultaneous detection across all three WWTPs was common (19 of 33 weeks with influenza detection; 57.6%), whereas detection in two or one WWTP was recorded in 5 or 9 weeks, respectively (Table 2). Influenza-related hospitalizations were more common during weeks with viral detection in wastewater (admissions in 12 of 33 weeks, 36.4%) than in weeks without influenza detection in wastewater (admissions in 4 of 14 weeks, 28.6%); however, this difference was not statistically significant (*p* = 0.87). Additionally, no significant correlation was found between the average weekly influenza virus concentration in WW and the number of influenza admissions (Spearman’s r = 0.02; *p* = 0.88). Interestingly, as with SARS-CoV-2, most weeks with influenza detection at all three plants clustered in a continuous period from week 40, 2024, through week 6, 2025 (13 of 19 weeks with widespread influenza detection, 68.4%) (Figure 2). The average weekly copy number of influenza in WW was also higher during this period than in the preceding period (weeks 24 to 39, 2024) and the subsequent period (weeks 7 to 24, 2025) (*p* < 0.001 for both comparisons). No difference was observed between the periods before and after this continuous widespread influenza detection (*p* = 0.94). Additionally, no positive influenza cases were recorded prior to the onset of widespread influenza circulation (weeks 25 to 39, 2024). In contrast, influenza hospitalizations occurred in 7 of 13 weeks during the period of widespread circulation (53.8%), totaling 24 hospitalizations. Hospitalizations were also common after the widespread detection of influenza in WW, with hospitalizations recorded in 9 of 18 weeks (50%) and a total of 15 hospitalizations. As with SARS-CoV-2, the correlation between influenza WW concentration and influenza-related hospitalizations increased with longer lag periods, reaching the highest correlations at a 9-week lag (Spearman’s r = 0.62; *p* = 0.0001) (Table S4).

RSV was detected in WW only in 13 weeks (27.7%). Simultaneous detection in all three WWTP occurred in only three weeks, while detection in only one WWTP was registered in 9 weeks (Table 2). RSV hospitalizations occurred more frequently in weeks in which this virus was detected in wastewater samples (9 of 13 weeks, 69.2%) than in weeks with no detection (11 of 34 weeks, 32.3%) (*p* = 0.022). However, a significant correlation between average RSV copy number in WW and the number of RSV hospitalizations was not observed (Spearman’s r = 0.10; *p* = 0.49). Interestingly, the higher frequency of hospitalizations was recorded during weeks in which RSV was detected in one or two WWTP (9 of 11 weeks, 81.8%), while no hospitalizations associated with this virus occurred during the three weeks in which RSV was detected in all three WWTP. Two of the three weeks with widespread RSV detection in WW were consecutive (weeks 40 and 41, 2024), coinciding with the onset of widespread SARS-CoV-2 and influenza detection. As noted for influenza, no RSV hospitalizations occurred during the period from weeks 24 to 40, 2024 (Figure 2). No RSV hospitalizations occurred during the two-week period in which widespread detection in wastewater was noted, and all admissions occurred thereafter (admissions in 20 of 29 weeks, 68.9%). Correlation analyses between RSV WW copy number and hospitalizations in subsequent weeks did not show a significant association with any lag time (Table S4).

Detection of hMPV in at least one WWTP was recorded in 40 weeks. Simultaneous detection at the three WWTPs occurred within 13 weeks, whereas detection at two plants was recorded within 14 weeks. This virus did not show a major period of widespread circulation, in contrast to the other three viruses. There were two periods of three consecutive weeks in which hMPV was detected in all three plants and two periods of two consecutive weeks in which hMPV was detected in the three plants. As a result, the study year was divided into five periods, defined by the three consecutive weeks of widespread viral circulation (Figure 2). No significant correlation between hMPV concentration in WW and the number of hospitalizations caused by this virus was observed (Spearman’s r = −0.06; *p* = 0.66). However, correlations with subsequent weeks showed a significant, albeit weak, correlation between WW hMPV copy number and hMPV-associated hospitalizations occurring three weeks later (Spearman’s r = 0.39; *p* = 0.009) (Table S4).

## 4. Discussion

Detection of SARS-CoV-2 and other respiratory viruses in WW samples has become an important epidemiological surveillance tool. For instance, the Centers for Disease Control and Prevention routinely reports WW surveillance data, along with other viral activity indicators [26]. Relevantly, there are important differences in detection patterns between viruses and across locations. As such, local data are required to establish the endemic corridor, which may enable better prediction of future outbreaks. In addition, interpretation of results requires the availability of clinical indicators, such as hospitalizations or deaths associated with specific pathogens. During the COVID-19 pandemic, a strong correlation between SARS-CoV-2 detection in WW and viral circulation was reported [27,28]. However, the degree of correlation varied across studies conducted in diverse regions and at different time points. As the pandemic evolved, the predictive ability of WW detection appeared to decrease [29].

Several factors, including the type of sample used for analysis, detection methods, and selected clinical or epidemiological endpoints, have an impact on the usefulness of this surveillance strategy [9,10]. Overall, we found little correlation between SARS-CoV-2 WW concentrations and the epidemiological behavior of this virus during the week, assessed by hospitalizations identified through a year-round surveillance system. In fact, SARS-CoV-2 was detected in WW in most weeks of the study period, while hospitalizations associated with this virus occurred during a more restricted time frame. Nevertheless, the fall and winter periods were characterized by higher concentrations of the virus in WW and by a period of continuous citywide spread. Notably, the onset of this high-circulation period occurred a few weeks before SARS-CoV-2 hospitalizations began to increase. As such, this feature (widespread, continuous circulation of the virus) might be a useful epidemiological marker to alert Public Health agencies regarding a potential increase in hospitalizations in the following weeks. These results are consistent with the findings of Medema et al. at the start of the COVID-19 pandemic in the Netherlands [30]. In their study, they detected SARS-CoV-2 in three out of seven WW sampling sites one week after the first COVID-19 case was identified in the Netherlands; WW samples obtained 10 days later showed widespread detection of SARS-CoV-2 (as shown by detection of this virus in all seven sampling sites), coinciding with an increase in the number of COVID-19 cases. Interestingly, in our study we found that analyses between WW SARS-CoV-2 detection and hospitalizations during subsequent weeks showed a progressive increase in correlation reaching a highly significant association for hospitalizations occurring 11 weeks after viral concentration measurements in WW (Spearman’s correlation coefficient = 0.59, *p* < 0.001); however we acknowledge that, given a low biological plausibility for this time lag, this finding may be spurious as the result of shared seasonality and multiple testing and, therefore, should not be interpreted as causally related. Additional data is needed for a more refined temporal analysis. On the one hand, the ongoing circulation of SARS-CoV-2 identified in our study may preclude precise prediction of community cases at this time; once an endemic corridor has been established (i.e., continued acquisition of WW epidemiological data for several years), changes in viral concentration in the water may enable prediction. On the other hand, we believe that in this post-pandemic scenario, where most of the population had the infection (often several times) and many subjects had had several dosages of SARS-CoV-2 vaccine, in addition to the circulation of less virulent strains, we may be looking at a sustained chain of transmission likely by asymptomatic shedders or people presenting mild symptoms not seeking for medical attention and presenting low risk of hospitalization, which was our comparative outcome. Moreover, viral shedding varies by strain, with higher levels for the ancestral and Iota strains; therefore, viral load quantification in WW and its correlation with community cases may vary with the circulating strain [31]

While our observations require confirmation in subsequent winter seasons, we observed similar results for influenza and, to a lesser extent, for RSV, with a period of continuous citywide circulation of these viruses preceding the onset of an increase in virus-associated hospitalizations. These results support the potential use of WW-based epidemiological surveillance for early identification of outbreaks of several respiratory viruses.

In contrast to SARS-CoV-2, which is currently used in many countries as part of routine surveillance, influenza and RSV detection is carried out in a more limited way [11,12,13,14]. Nevertheless, available information indicates that WW-based surveillance may be a useful tool to guide recommendations by Public Health authorities. For instance, a study conducted in Ontario showed that RSV WW-based surveillance could inform recommendations for the application of monoclonal antibodies against this virus [32]. However, it is worth noting that WW-based surveillance requires local epidemiological and disease activity information in order to interpret the results obtained from WW samples to define the potential application. Although RSV was detected least frequently in WW in the present study, widespread detection in WW was identified shortly before the surge in RSV hospitalizations. These observations highlight the potential use of WW-based surveillance to identify the onset of a high risk of viral transmission.

Influenza viruses were also frequently detected in WW samples. No significant association between viral concentration in WW and influenza hospital admissions in the same week was observed, but a positive correlation was observed with increasing lag times from 4 to 9 weeks. However, at this time, we cannot establish a fixed period for predicting future severe influenza surges based on WW concentrations, as many factors may affect viral circulation and hospitalization rates, as well as the previously mentioned limitation of our data to perform a refined temporal analysis and discard spurious correlations. It is well known that vaccine efficacy varies from season to season, and the effectiveness observed during our surveillance year may differ in subsequent seasons, thereby altering hospitalization rates [33]. Moreover, it should be considered that WW surveillance may be more suitable for some viruses than others, as viral fecal excretion levels vary between viruses (i.e., higher for SARS-CoV-2 than influenza or RSV), and there may be variations for different types or subtypes of influenza viruses, which may affect the predictive ability of WW detection [34]. In addition, the shedding duration varies across viruses, reducing detection in WW and thus their efficacy as potential predictors of outbreaks. Notwithstanding, viral detection in WW during periods of low clinically evident influenza infections is an interesting finding that suggests that this virus may be more prevalent outside of epidemic periods than usually thought. This could also be explained by the detection of influenza viruses from other species in WW, as suggested by detection of H5N1 viruses in Texas in the absence of human infections associated with this influenza subtype [35]. Although the influenza assays used in this study do not distinguish between human and non-human influenza A viruses, additional testing of a subset of positive samples for avian influenza A H5 and H7 was negative, and no avian influenza cases were reported locally during the study period. These observations support a predominantly human origin of the influenza signals detected in wastewater, although a minor contribution of non-human influenza A viruses cannot be completely excluded.

The higher detection rate for hMPV compared with RSV was surprising, since RSV is usually associated with a higher number of infections than hMPV, particularly in children. Nevertheless, after the SARS-CoV-2 pandemic an increase in the detection of hMPV has been reported in several studies. In the present study, we detected hMPV in a higher number of children (199/330; 36%) than during the 2023–2024 season at the same hospital (68/390; 17.4%) [24]. This proportion was also higher than the number of detections reported prior to the pandemic in a study carried out in several hospitals in Mexico (112/1404; 7.98%) [36]. Continuous surveillance (both in clinical and wastewater samples) will allow us to assess whether this pattern persists during subsequent seasons. It was also notable that no RSV hospitalizations occurred during weeks in which RSV was detected in the three WWTP. The reason for this result is unclear, but may be explained, in part, by the short continuous period in which RSV was detected in all WWTP.

A limitation of our study is that we had no WW samples available for six weeks due to administrative constraints at the WWTPs. Nevertheless, we confirmed the potential predictive ability of WW surveillance across diverse viruses. This was possible, in part, due to the year-round epidemiological surveillance at the hospital, which enabled consistent measurement of a virological endpoint throughout the study period and for lag correlations between WW and clinical data. Another strength of the study is the inclusion of several WWTPs that cover most of the population of the metropolitan area of San Luis Potosí. Notably, a significant statistical correlation was observed across most WW viral concentrations at the three WWTPs; future studies may assess the optimal number of samples or WWTPs required for surveillance programs. Nevertheless, despite high correlations among WWTPs, differences were observed, and in many weeks, a virus was detected only in one WWTP. In addition, our results suggest that, in addition to viral concentrations, the identification of continuous, widespread circulation of a virus (i.e., detection of the virus during consecutive weeks at all WWTPs) may be useful as an early warning of increased hospitalizations. Another limitation of this study is that sample-specific recovery efficiencies were not calculated, and viral concentrations were not corrected for recovery losses. Nevertheless, preliminary spiking experiments and the systematic use of crAssphage as a fecal and process quality control support the robustness of the detection results across samples and time. Also, the use of a single pediatric center as reference for the clinical impact of respiratory viruses may not be representative of the entire population. Unfortunately, no other systematic year-round clinical and virological information for RSV, influenza, SARS-CoV-2, and hMPV was available, since routine viral detection of all hospitalized patients (pediatric or adult) is not carried out in Mexico. As a result, in most pneumonia cases, even in severe instances, the specific pathogen causing the illness is not identified [37]. Nevertheless, the weekly number of pediatric hospital admissions in our study correlated with the number of pneumonia cases reported in San Luis Potosí during the study period.

In conclusion, WW surveillance is feasible and useful in middle-income countries. However, the predictive value may vary according to several factors (virus fecal shedding, the presence of more virulent strains, seasonality, and the simultaneous presence of other viruses). As such, the potential use as an “early warning” of the results of a surveillance system as presented here would be qualitative and requires evaluation of sensitivity, positive predictive value, and false-alarm rates. To this end, a longer surveillance period is required to provide data on subsequent epidemics to test whether the proposed widespread circulation definition, or another measurement, could adequately inform Public Health interventions. Establishing endemic corridors for each virus at a given location may improve the predictive value of WW epidemiology and should always be accompanied by well-established population surveillance programs to assess its efficacy.

## Figures and Tables

**Table 1:** Frequency of SARS-CoV-2, influenza, RSV, and hMPV detection in wastewater samples in San Luis Potosí, Mexico (2024–2025).

	WW-P1	WW-P2	WW-N	All Samples
SARS-CoV-2				
Detection (samples)	53/81 (65.4%)	49/80 (61.2%)	50/77 (64.9%)	152/238 (63.9%)
Copies per sample (log 10 (SARS-CoV-2/crAssphage ratio)), mean (SD)	1.62 (1.55)	1.57 (1.66)	1.72 (1.54)	1.64 (1.59)
Detection (weeks)	37/47 (78.7%)	32/47 (68.1%)	35/47 (74.5%)	42/47 (89.4%)
Copies per week (log 10 (SARS-CoV-2/crAssphage ratio)), mean (SD)	2.07 (1.44)	1.94 (1.64)	1.99 (1.48)	2.35 (1.28) *
Influenza				
Detection (samples)	38/81 (46.9%)	32/80 (40%)	38/77 (49.3%)	108/238 (45.4%)
Copies per sample (log 10 (SARS-CoV-2/crAssphage ratio)), mean (SD)	1.11 (1.37)	0.92 (1.37)	1.13 (1.36)	1.05 (1.37)
Detection (weeks)	26/47 (55.3%)	25/47 (53.2%)	25/47 (53.2%)	33/47 (70.2%)
Copies per week (log 10 (SARS-CoV-2/crAssphage ratio)), mean (SD)	1.42 (1.43)	1.20 (1.47)	1.29 (1.41)	1.61 (1.40) *
RSV				
Detection (samples)	7/81 (8.6%)	9/80 (11.2%)	8/77 (10.4%)	24/238 (10.1%)
Copies per sample (log 10 (SARS-CoV-2/crAssphage ratio)), mean (SD)	0.31 (1.04)	0.28 (0.93)	0.003 (0.02)	0.19 (0.82)
Detection (weeks)	6/47 (12.8%)	7/47 (14.9%)	7/47 (14.9%)	13/47 (27.7%)
Copies per week (log 10 (SARS-CoV-2/crAssphage ratio)), mean (SD)	0.43 (1.17)	0.40 (1.08)	0.003 (0.02)	0.47 (1.03) *
hMPV				
Detection (samples)	35/81 (43.2%)	32/80 (40%)	28/77 (36.4%)	95/238 (39.9%)
Copies per sample (log 10 (SARS-CoV-2/crAssphage ratio)), mean (SD)	1.22 (1.60)	1.13 (1.63)	0.02 (0.12)	0.79 (1.43)
Detection (weeks)	29/47 (61.7%)	27/47 (57.4%)	24/47 (51.1%)	40/47 (85.1%)
Copies per week (log 10 (SARS-CoV-2/crAssphage ratio)), mean (SD)	1.72 (1.67)	1.62 (1.69)	0.02 (0.12)	1.70 (1.36) *

* Weighted mean based on flow rate.

**Table 2:** Correlation of weekly viral detections in WW collected from three WWTP in San Luis Potosí, Mexico.

	Correlation Between P1-WWTP and P2-WWTP	Correlation Between P2-WWTP and PN-WWTP	Correlation Between PN-WWTP and P1-WWTP
SARS-CoV-2	rs = 0.63 (*p* < 0.001)	rs = 0.63 (*p* < 0.001)	rs = 0.52 (*p* < 0.001)
Influenza	rs = 0.64 (*p* < 0.001)	rs = 0.73 (*p* < 0.001)	rs = 0.62 (*p* < 0.001)
RSV	rs = 0.47 (*p* = 0.001)	rs = 0.99 (*p* < 0.001)	rs = 0.46 (*p* = 0.001)
hMPV	rs = 0.33 (*p* = 0.026)	rs = 0.98 (*p* < 0.001)	rs = 0.34 (*p* = 0.021)

**Table 3:** Frequency of hospitalizations associated with respiratory viruses according to the number of WWTP in which each virus was detected in a week.

	Detection in 0 WWTP	Detection in 1 WWTP	Detection in 2 WWTP	Detection in 3 WWTP	Detection in any WWTP	Total Period
SARS-CoV-2						
Weeks with admissions/number of weeks based on WW results	3/5 (60%)	3/5 (60%)	5/12 (41.7%)	10/25 (40%)	18 42 (44.2%)	21/47 (44.7%)
Number of SARS-CoV-2 admissions	11	6	20	41	67	78
Average SARS-CoV-2 admissions per week	2.2	1.2	1.67	1.64	1.59	1.66
Influenza						
Weeks with admissions/number of weeks based on WW results	4/14 (28.6%)	2/9 (22.2%)	1/5 (20%)	9/19 (47.4%)	12/33 (36.4%)	16/47 (34%)
Number of influenza admissions	7	4	1	27	32	39
Average influenza admissions per week	0.5	0.44	0.2	1.42	0.97	0.83
RSV						
Weeks with admissions/number of weeks based on WW results	12/34 (35.3%)	7/9 (77.8%)	1/1 (100%)	0/3 (0%)	8/13 (61.5%)	20/47 (42.5%)
Number of RSV admissions	63	50	2	0	52	115
Average RSV admissions per week	1.85	5.55	2	0	4	2.45
hMPV						
Weeks with admissions/number of weeks based on WW results	2/7 (28.6%)	5/13 (38.5%)	7/14 (50%)	4/13 (30.8%)	16/40 (40%)	18/47 (38.3%)
Number of hMPV admissions	6	22	53	14	89	95
Average hMPV admissions per week	0.86	1.69	3.78	1.08	2.22	2.02

## Data Availability

Data used for this study will be available at CICSsB’s Wastewater Surveillance Project webpage: https://www.uaslp.mx/CICSaB/Paginas/Laboratorio-Enfermedades-Infecciosas-/9237#gsc.tab=0, accessed 5 January 2026.

## Supplementary Materials

The following supporting information can be downloaded at: https://www.mdpi.com/article/10.3390/v18020254/s1, Figure S1. Weekly numbers of hospital admissions for acute respiratory infections at Hospital de la Niñez y la Mujer “Dr. Alberto López Hermosa and pneumonia cases reported in San Luis Potosí by the Health Ministry between epidemiological week 24, 2024 and epidemiological week 24, 2025; Table S1: Final concentrations of reagents used in qPCR assays for the detection of SARS-CoV-2, RSV, hMPV, and Influenza A/B. Final or reference concentrations of the mix and forward and reverse primers are shown; Table S2: Thermal cycling protocols used for quantitative PCR (qPCR) detection of SARS-CoV-2, RSV, hMPV, and Influenza A/B; Table S3: Primer sequences used for qPCR detection of respiratory viruses, including the targeted genomic region and reference source; Table S4: Correlation between virus detection in WW in a given week and the number of hospitalizations in which that virus was detected in the same or subsequent (lag) weeks.

## Abbreviations

The following abbreviations are used in this manuscript:

WW	Wastewater
WWTP	Wastewater treatment plant
SARS-CoV-2	Severe acute respiratory syndrome coronavirus 2
RSV	Respiratory syncytial virus
hMPV	Human metapneumovirus
COVID-19	Coronavirus disease 2019
