# Supplementary material for: Wastewater-Based Surveillance of Respiratory Viruses in the SARS-CoV-2 Post-Pandemic Period in Mexico
Source: Viruses. 2026 Feb 17;18(2):254. doi: 10.3390/v18020254 (PMC12945105; doi:10.3390/v18020254)
Supplement: Supplementary file 1 [file viruses-18-00254-s001.zip › viruses-4115903-supplementary.pdf]

SUPPLEMENTARY FIGURES AND TABLES

Figure S1. Weekly numbers of hospital admissions for acute respiratory infections at Hospital de la Niñez y la Mujer "Dr. Alberto López Hermosa and pneumonia cases reported in San Luis Potosí by the Health Ministry between epidemiological week 24, 2024 and epidemiological week 24, 2025.

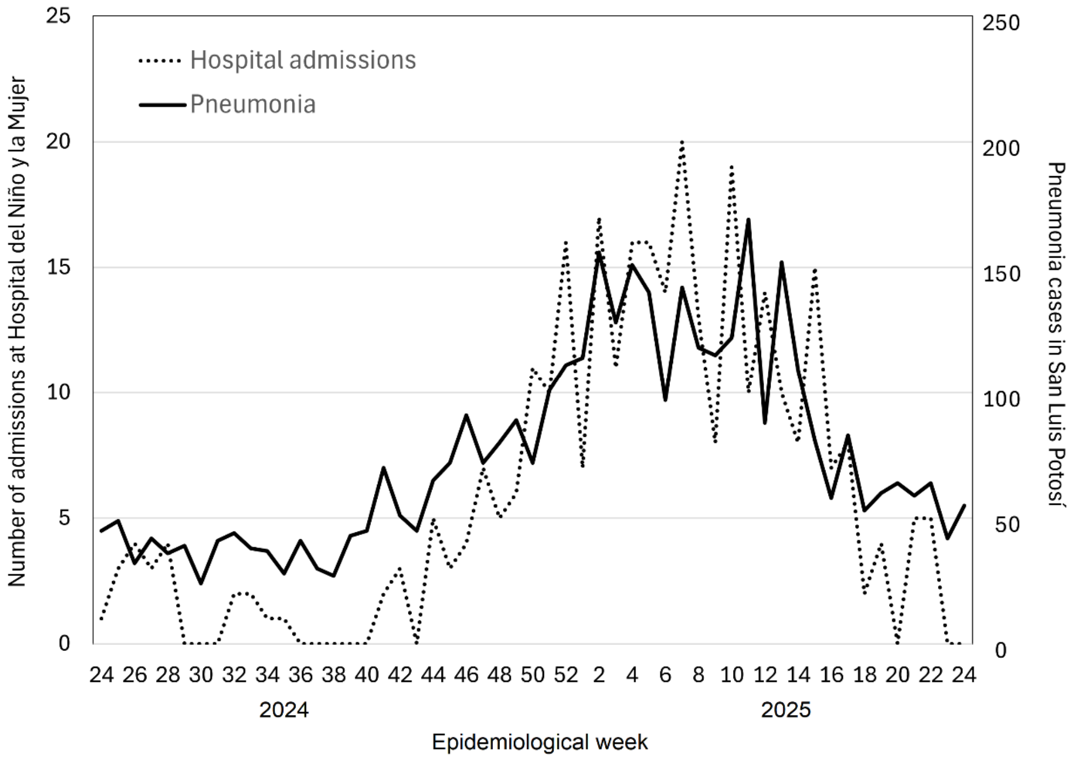

Table S1. Final concentrations of reagents used in qPCR assays for the detection of SARS-CoV-2, RSV, hMPV, and Influenza A/B. Final or reference concentrations of the mix and forward and reverse primers are shown.

**Reagent**	**SARS-CoV-2**	**RSV**	**hMPV**	**Influenza A/B**	**Influenza H5/H7**	**CrAssphage**
Maxima SYBR Green/ROX Master Mix	1×	1×	1×	1×	1×	1×
Forward primer	0.3 µM	0.3 µM	0.3 µM	0.3 µM	0.3 µM	0.3 µM
Reverse primer	0.3 µM	0.3 µM	0.3 µM	0.3 µM	0.3 µM	0.3 µM
Nuclease-free water	—	—	—	—	—	—
cDNA template	—	—	—	—	—	—

Note: Dashes indicate variable volumes adjusted to reach the final reaction volume.

Table S2. Thermal cycling protocols used for quantitative PCR (qPCR) detection of SARS-CoV-2, RSV, hMPV, and Influenza A/B.

Virus	Step	Temperature	Time	Cycles
**SARS-CoV-2**	Initial denaturation	95 °C	5 min	1
	Denaturation	95 °C	30 s	45
	Annealing	59 °C	30 s	
	Extension	72 °C	30 s	
**RSV**	Initial denaturation	95 °C	10 min	1
	Denaturation	95 °C	15 s	40
	Annealing	54.5 °C	30 s	
	Extension	72 °C	30 s	
**hMPV**	Initial denaturation	95 °C	10 min	1
	Denaturation	95 °C	15 s	40
	Annealing	55 °C	30 s	
	Extension	72 °C	30 s	
**Influenza A/B**	Initial denaturation	95 °C	10 min	1
	Denaturation	95 °C	15 s	40
	Annealing	60°C	30 s	
	Extension	72 °C	30 s	
**Influenza H5/H7**	Initial denaturation	95 °C	10 min	1
	Denaturation	95 °C	15 s	40
	Annealing	60°C	30 s	
	Extension	72 °C	30 s	
**CrAssphage**	Initial denaturation	95 °C	10 min	1
	Denaturation	95 °C	15 s	40
	Annealing	60°C	30 s	
	Extension	72 °C	30 s	

Table S3. Primer sequences used for qPCR detection of respiratory viruses, including the targeted genomic region and reference source.

Virus	Target gene/region	Primer sequence (5′–3′)	Reference
**SARS-CoV-2**	Nucleocapsid (N)	Forward: GACCCCAAAATCAGCGAAAT<br>Reverse: TCTGGTTACTGCCAGTTGAATCTG	Lu et al., 2020
**Influenza A**	Matrix (M2)	Forward: ATGAGYCTTYTAACCGAGGTCGAAACG<br>Reverse: TGGACAAANCGTCTACGCTGCAG	Terrier et al., 2011
**Influenza B**	Hemagglutinin (HA)	Forward: AGACCAGAGGGAAACTATGCCC<br>Reverse: TCCGGATGTAACAGGTCTGACTT	van Elden et al., 2001
**RSV**	Intergenic region between phosphoprotein (P) and matrix (M) genes	Forward: GCAAATATGGAAACATACGTGAA<br>Reverse: TCTTTTTCTAGGACATTGTAYTGAACA	Lara-Hernández et al., 2023
**hMPV**	Nucleocapsid (N)	Forward: TGATGCRCTCAAAAGATACCC<br>Reverse: GCAAAGCAGAAAGTTTRTTYGT	Martínez-Marrero et al., 2025
**Influenza H5**	Hemagglutinin (HA)	Forward: CARGGGAGTGGDTAYGCBGCAGA<br>Reverse: ARAAGTTCAGCRTTRTARGTCCA	World Health Organization, 2021
**Influenza H7**	Hemagglutinin (HA)	Forward: AGAAATGAAATGGCTCCTGTCAA<br>Reverse: GGTTTTTTCTTGTATTTTTATATGACTTAG	World Health Organization, 2021
**CrAssphage**	Genomic region 14731-14856	Forward: CAGAAGTACAAACTCCTAAAAAACGTAGAG<br>Reverse: GATGACCAATAAACAAGCCATTAGC	Stachler et al., 2020

1. Lu, X.; Wang, L.; Sakthivel, S.K.; Whitaker, B.; Murray, J.; Kamili, S.; Lynch, B.; Malapati, L.; Burke, S.A.; Harcourt, J.; et al. US CDC Real-Time Reverse Transcription PCR Panel for Detection of Severe Acute Respiratory Syndrome Coronavirus 2. *Emerg Infect Dis* **2020**, *26*, 1654–1665, doi:10.3201/eid2608.201246.
2. Terrier, O.; Josset, L.; Textoris, J.; Marcel, V.; Cartet, G.; Ferraris, O.; N'Guyen, C.; Lina, B.; Diaz, J.J.; Bourdon, J.C.; et al. Cellular transcriptional profiling in human lung epithelial cells infected by different subtypes of influenza A viruses reveals an overall down-regulation of the host p53 pathway. *Virol J* **2011**, *8*, 285, doi:10.1186/1743-422X-8-285.
3. van Elden, L.J.; Nijhuis, M.; Schipper, P.; Schuurman, R.; van Loon, A.M. Simultaneous detection of influenza viruses A and B using real-time quantitative PCR. *J Clin Microbiol* **2001**, *39*, 196–200, doi:10.1128/JCM.39.1.196-200.2001.
4. Lara-Hernandez, I.; Munoz-Escalante, J.C.; Bernal-Silva, S.; Noyola, D.E.; Wong-Chew, R.M.; Comas-Garcia, A.; Comas-Garcia, M. Ultrastructural and Functional Characterization of Mitochondrial Dynamics Induced by Human Respiratory Syncytial Virus Infection in HEp-2 Cells. *Viruses* **2023**, *15*, doi:10.3390/v15071518.
5. Martinez-Marrero, N.; Munoz-Escalante, J.C.; Yerena-Rivera, J.M.; Jaime-Rocha, L.R.; Leija-Martinez, J.J.; Gonzalez-Ortiz, A.M.; Noyola, D.E. Molecular Epidemiology of Human Metapneumovirus Infections in Children from San Luis Potosi-Mexico. *Viruses* **2025**, *17*, doi:10.3390/v17101338.
6. World Health Organization. WHO information for the molecular detection of influenza viruses. World Health Organization, Geneva, 2024. Available at: https://cdn.who.int/media/docs/default-source/influenza/molecular-detention-of-influenza-viruses/protocols_influenza_virus_detection_2024.pdf?sfvrsn=df7d268a_8 ; Accessed: February 1, 2026.
7. Stachler, E., Kelty, C., Sivaganesan, M., Li, X., Bibby, K., & Shanks, O. C. (2017). Quantitative CrAssphage PCR assays for human fecal pollution measurement. Environmental science & technology, 51(16), 9146-9154.

Table S4. Correlation between virus detection in WW in a given week and the number of hospitalizations in which that virus was detected in the same or subsequent (lag) weeks

Week of admission with which comparisons were made	SARS-CoV-2	Influenza	RSV	hMPV
Same week	r_s = -0.22, p = 0.13	r_s = 0.02, p = 0.88	r_s = 0.10, p = 0.49	r_s =-0.06, p = 0.66
Lag 1	r_s = -0.16, p = 0.29	r_s = 0.29, p = 0.05	r_s = 0.19, p = 0.23	r_s = 0.23, p = 0.13
Lag2	r_s = -0.01, p = 0.94	r_s = 0.26, p = 0.09	r_s = 0.30, p = 0.05	r_s = 0.27, p = 0.08
Lag3	r_s = -0.02, p = 0.89	r_s = 0.20, p = 0.19	r_s = 0.19, p = 0.21	r_s = 0.39, p = 0.009
Lag4	r_s = 0.07, p = 0.68	r_s = 0.31, p = 0.04	r_s = 0.09, p = 0.58	r_s = 0.24, p = 0.12
Lag5	r_s = 0.05, p = 0.75	r_s = 0.35, p = 0.03	r_s = 0.08, p = 0.61	r_s = 0.12, p = 0.44
Lag6	r_s = 0.24, p = 0.14	r_s = 0.44, p = 0.005	r_s = 0.14, p = 0.38	r_s = 0.13, p = 0.43
Lag7	r_s = 0.31, p = 0.05	r_s = 0.52, p <0.001	r_s = 0.20, p = 0.22	r_s = 0.09, p = 0.59
Lag8	r_s = 0.29, p = 0.07	r_s = 0.39, p = 0.01	r_s = 0.18, p = 0.28	r_s = 0.11, p = 0.50
Lag9	r_s = 0.32, p = 0.06	r_s = 0.62, p = 0.0001	r_s = 0.11, p = 0.49	r_s = 0.31, p = 0.06

Lag10	$r_s = 0.29$, $p = 0.08$	$r_s = 0.55$, $p < 0.001$	$r_s = 0.03$, $p = 0.87$	$r_s = 0.14$, $p = 0.40$
Lag11	$r_s = 0.61$, $p = 0.002$	$r_s = 0.47$, $p = 0.004$	$r_s = 0.06$, $p = 0.71$	$r_s = 0.13$, $p = 0.47$
Lag12	$r_s = 0.63$, $p = 0.001$	$r_s = 0.61$, $p < 0.001$	$r_s = 0.07$, $p = 0.70$	$r_s = 0.01$, $p = 0.97$
Lag13	$r_s = 0.49$, $p = 0.004$	$r_s = 0.34$, $p = 0.05$	$r_s = 0.03$, $p = 0.85$	$r_s = 0.02$, $p = 0.89$
Lag14	$r_s = 0.39$, $p = 0.03$	$r_s = 0.26$, $p = 0.15$	$r_s = 0.04$, $p = 0.81$	$r_s = -0.09$, $p = 0.63$
